# Young Person and Parents/Carers' Experiences of the Transition Into a Child and Adolescent Mental Health Services (CAMHS) Inpatient Unit: What Could Be Improved?

**DOI:** 10.1192/bjo.2024.381

**Published:** 2024-08-01

**Authors:** Georgina Shajan, Ashika Shah, Paul Briley, Pallab Majumder, Josephine Holland

**Affiliations:** 1 University of Nottingham, Nottingham, United Kingdom; 2 Nottinghamshire Healthcare NHS Foundation Trust, Nottingham, United Kingdom

## Abstract

**Aims:**

To investigate the themes within young people and parents/carers’ experiences of the admission process. A focus was placed on potential targets for change to improve experiences of CAMHS admission.

**Methods:**

Young people and parents/carers with an experience of inpatient CAMHS admission within the past two years were approached by the Involvement team of an NHS trust. Focus groups and interviews were conducted capturing the views of 8 young people and two parents/carers. The notes and transcripts from these conversations were analysed using Braun and Clarke thematic analysis.

**Results:**

Two key themes were identified within the data. The first focussed on information provision and communication. This captured young people's experiences of both: what information was available to them, e.g., websites and leaflets, and how this information was conveyed to them. The second theme brought together the young people's interpersonal experiences during the admission process. Within this, the impact of consistent contacts as well as both positive and negative transient encounters was highlighted.

**Conclusion:**

Admission to a psychiatric ward is often a highly distressing time for young people and their families. The provision of easily accessible, clear, and correct information can improve their expectations and initial impressions of a unit. How this information is presented is also important. Consistent staff support and response to distress and difficulties during this time can also shape the perspectives of young people and their parents/carers.

Clear, accurate, and young person friendly information about a unit and the admissions process could be an easily achievable change which units could make to improve young person experiences. Improvements to clinicians’ skills and response may represent a more complex and expensive goal.

